# Poly[bis­(acetonitrile-κ*N*)di-μ-thio­cyanato-κ^2^ *N*,*S*;κ^2^ *S*,*N*-nickel(II)]

**DOI:** 10.1107/S1600536811004132

**Published:** 2011-02-05

**Authors:** Susanne Wöhlert, Inke Jess, Christian Näther

**Affiliations:** a Institut für Anorganische Chemie, Christian-Albrechts-Universität Kiel, Max-Eyth-Strasse 2, 24098 Kiel, Germany

## Abstract

In the title compound, [Ni(NCS)_2_(CH_3_CN)_2_]_*n*_, the Ni^II^ cation is coordinated by two *N*-bonded and two *S*-bonded thio­cyanate anions, as well as two acetonitrile mol­ecules in an octa­hedral NiN_4_S_2_ coordination mode. The asymmetric unit comprises one nickel cation, two thio­cyanate anions and two actonitrile mol­ecules. In the crystal, the Ni^II^ cations are connected by bridging thio­cyanate anions into a three-dimensional coordination network.

## Related literature

For background of this work see: Boeckmann & Näther (2010 ▶); Wriedt *et al.* (2009*a* ▶,*b* ▶).
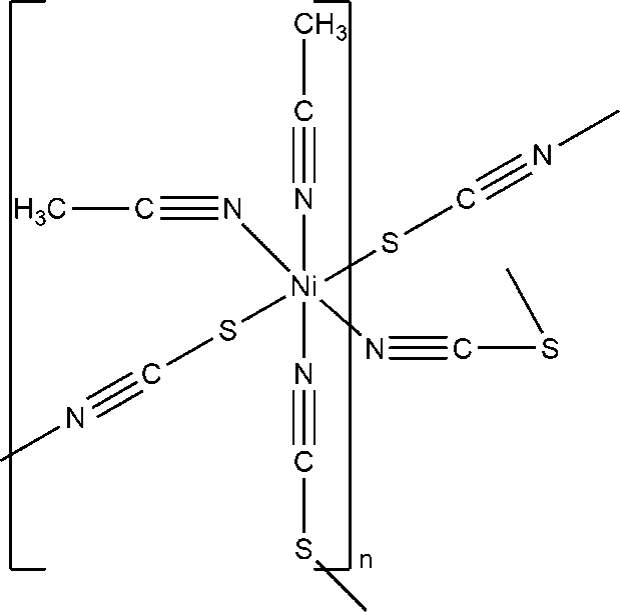


## Experimental

### 

#### Crystal data

[Ni(NCS)_2_(C_2_H_3_N)_2_]
*M* *_r_* = 256.98
Orthorhombic, 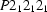

*a* = 9.0666 (4) Å
*b* = 9.1215 (3) Å
*c* = 12.0696 (6) Å
*V* = 998.17 (7) Å^3^
*Z* = 4
Mo *K*α radiation
μ = 2.32 mm^−1^
*T* = 293 K
0.11 × 0.09 × 0.06 mm

#### Data collection

Stoe IPDS-2 diffractometer
Absorption correction: numerical (*X-SHAPE* and *X-RED32*; Stoe & Cie, 2008) ▶ *T*_min_ = 0.683, *T*_max_ = 0.772
11157 measured reflections
2694 independent reflections
2479 reflections with *I* > 2σ(*I*)
*R*_int_ = 0.023

#### Refinement

*R*[*F* ^2^ > 2σ(*F* ^2^)] = 0.027
*wR*(*F* ^2^) = 0.051
*S* = 1.29
2694 reflections
120 parameters
H-atom parameters constrained
Δρ_max_ = 0.29 e Å^−3^
Δρ_min_ = −0.28 e Å^−3^
Absolute structure: Flack (1983 ▶), 1141 Friedel pairs
Flack parameter: −0.003 (13)

### 

Data collection: *X-AREA* (Stoe & Cie, 2008) ▶; cell refinement: *X-AREA* ▶; data reduction: *X-AREA*; program(s) used to solve structure: *SHELXS97* (Sheldrick, 2008 ▶); program(s) used to refine structure: *SHELXL97* (Sheldrick, 2008) ▶; molecular graphics: *XP* in *SHELXTL* (Sheldrick, 2008 ▶) and *DIAMOND* (Brandenburg, 1999 ▶); software used to prepare material for publication: *XCIF* in *SHELXTL*.

## Supplementary Material

Crystal structure: contains datablocks I, global. DOI: 10.1107/S1600536811004132/im2264sup1.cif

Structure factors: contains datablocks I. DOI: 10.1107/S1600536811004132/im2264Isup2.hkl

Additional supplementary materials:  crystallographic information; 3D view; checkCIF report

## Figures and Tables

**Fig. 1.**
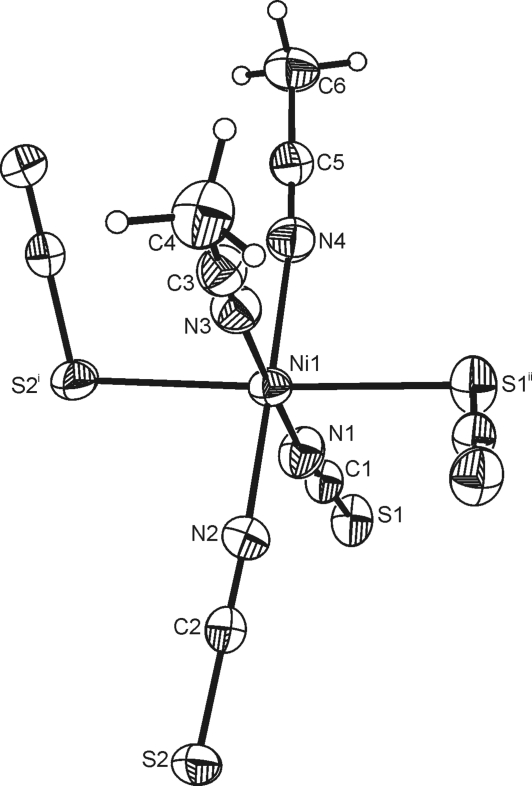
Crystal structure of the title compound with labelling and displacement ellipsoids drawn at the 30 % probability level. Symmetry codes: i = x-1/2, -y+3/2, -z+1; ii = -x, y-1/2, -z+3/2.

**Fig. 2.**
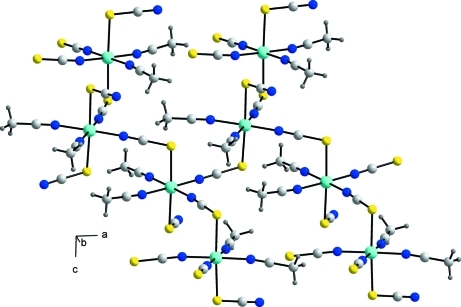
Crystal structure of the title compound approximately viewed along the crystallographic b-axis.

## supplementary crystallographic information

### Comment

In recent work, we have shown that thermal decomposition reactions are an elegant route for discovering and synthesising new ligand-deficient coordination polymers with attractive magnetic properties (Boeckmann & Näther, 2010; Wriedt *et al.*, 2009*a*, 2009*b*). In our investigation on the syntheses, structures and properties of such compounds based on paramagnetic transition metals, pseudo-halides and N-donor ligands, we have reacted nickel(II) thiocyanate and *trans*-1,2-bis(4-pyridyl)-ethylene in acteonitrile. In this reaction single crystals of the title compound were obtained accidentally in a mixture with an unknown phase. To identify the reaction product the compound was investigated by single crystal X-ray diffraction.

In the crystal structure of the title compound, each nickel(II) cation is coordinated by four bridging thiocyanato anions and by two acetonitrile molecules (Fig. 1). The NiN_4_S_2_ octahedron is slightly distorted with two long Ni—SCN distances of 2.5305 (6) Å and 2.5341 (6) Å as well as two short Ni—NCS distances of 2.021 (2) Å and 2.023 (2) Å. The angles around the metal atom range from 87.88 (6) ° to 93.23 (6) ° and 178° (Tab. 1).

The nickel cations are linked by the thiocyanato anions into chains, that are further connected into a three-dimensional network (Fig. 2). The shortest intramolecular Ni···Ni distance amounts to 5.7052 (4) Å and the shortest intermolecular Ni···Ni distance amounts to 9.0666 (4) Å.

### Experimental

Ni(NCS)_2_ was obtained from Alfa Aesar and *trans*-1,2-bis(4-pyridyl)-ethylene (bpe) was obtained from Sigma Aldrich. All chemicals were used without further purification. 0.6 mmol (104.7 mg) Ni(NCS)_2_ and 0.15 mmol (28.2 mg) bpe were reacted with 1 ml acetonitrile in a closed test-tube at 120°C for three days. On cooling blue block-shaped single crystals of the title compound were obtained in a mixture with a unknown phase. It must be noted, that the reaction without bpe does not lead to the formation of the title compound.

### Refinement

H atoms were positioned with idealized geometry, allowed to rotate but not to tip and were refined isotropically with *U*_iso_(H) = 1.5*U*_eq_(C) and C—H distances of 0.96 Å using a riding model. The absolute structure was determined on the basis of 1127 Friedel pairs.

### Crystal data

**Table:**

[Ni(NCS)_2_(C_2_H_3_N)_2_]	*F*(000) = 520
*M**_r_* = 256.98	*D*_x_ = 1.710 Mg m^−^^3^
Orthorhombic, *P*2_1_2_1_2_1_	Mo *K*α radiation, λ = 0.71073 Å
Hall symbol: P 2ac 2ab	Cell parameters from 11157 reflections
*a* = 9.0666 (4) Å	θ = 2.8–29.2°
*b* = 9.1215 (3) Å	µ = 2.32 mm^−^^1^
*c* = 12.0696 (6) Å	*T* = 293 K
*V* = 998.17 (7) Å^3^	Block, blue
*Z* = 4	0.11 × 0.09 × 0.06 mm

### Data collection

**Table:**

Stoe IPDS-2 diffractometer	2694 independent reflections
Radiation source: fine-focus sealed tube	2479 reflections with *I* > 2σ(*I*)
graphite	*R*_int_ = 0.023
ω scans	θ_max_ = 29.2°, θ_min_ = 2.8°
Absorption correction: numerical (*X-SHAPE* and *X-RED32*; Stoe & Cie, 2008)	*h* = −12→10
*T*_min_ = 0.683, *T*_max_ = 0.772	*k* = −12→12
11157 measured reflections	*l* = −16→16

### Refinement

**Table:**

Refinement on *F*^2^	Secondary atom site location: difference Fourier map
Least-squares matrix: full	Hydrogen site location: inferred from neighbouring sites
*R*[*F*^2^ > 2σ(*F*^2^)] = 0.027	H-atom parameters constrained
*wR*(*F*^2^) = 0.051	*w* = 1/[σ^2^(*F*_o_^2^) + (0.0221*P*)^2^] where *P* = (*F*_o_^2^ + 2*F*_c_^2^)/3
*S* = 1.29	(Δ/σ)_max_ = 0.001
2694 reflections	Δρ_max_ = 0.29 e Å^−^^3^
120 parameters	Δρ_min_ = −0.28 e Å^−^^3^
0 restraints	Absolute structure: Flack (1983), 1127 Friedel pairs
Primary atom site location: structure-invariant direct methods	Flack parameter: −0.003 (13)

### Special details

**Table:**

Geometry. All esds (except the esd in the dihedral angle between two l.s. planes) are estimated using the full covariance matrix. The cell esds are taken into account individually in the estimation of esds in distances, angles and torsion angles; correlations between esds in cell parameters are only used when they are defined by crystal symmetry. An approximate (isotropic) treatment of cell esds is used for estimating esds involving l.s. planes.
Refinement. Refinement of F^2^ against ALL reflections. The weighted R-factor wR and goodness of fit S are based on F^2^, conventional R-factors R are based on F, with F set to zero for negative F^2^. The threshold expression of F^2^ > 2sigma(F^2^) is used only for calculating R-factors(gt) etc. and is not relevant to the choice of reflections for refinement. R-factors based on F^2^ are statistically about twice as large as those based on F, and R- factors based on ALL data will be even larger.

### Fractional atomic coordinates and isotropic or equivalent isotropic displacement parameters (Å^2^)

**Table:**

	*x*	*y*	*z*	*U*_iso_*/*U*_eq_	
Ni1	−0.10172 (3)	0.83322 (3)	0.62898 (2)	0.02615 (6)	
N1	−0.0070 (2)	1.0335 (2)	0.63730 (18)	0.0388 (4)	
C1	0.0391 (2)	1.1504 (3)	0.64845 (15)	0.0307 (4)	
S1	0.10459 (8)	1.31745 (6)	0.66142 (4)	0.03781 (12)	
N2	0.0983 (2)	0.7362 (2)	0.61691 (15)	0.0356 (4)	
C2	0.2172 (2)	0.6972 (2)	0.60194 (15)	0.0283 (4)	
S2	0.38867 (6)	0.64753 (7)	0.58006 (4)	0.03776 (13)	
N3	−0.2036 (2)	0.6293 (2)	0.62176 (17)	0.0341 (4)	
C3	−0.2491 (2)	0.5139 (3)	0.6242 (2)	0.0335 (4)	
C4	−0.3109 (3)	0.3669 (3)	0.6278 (3)	0.0468 (6)	
H4A	−0.4134	0.3723	0.6472	0.070*	
H4B	−0.2593	0.3098	0.6823	0.070*	
H4C	−0.3007	0.3215	0.5565	0.070*	
N4	−0.3065 (2)	0.9326 (2)	0.63801 (17)	0.0365 (4)	
C5	−0.4170 (2)	0.9893 (2)	0.63197 (19)	0.0344 (4)	
C6	−0.5579 (3)	1.0633 (3)	0.6230 (3)	0.0452 (5)	
H6A	−0.5743	1.1215	0.6881	0.068*	
H6B	−0.6351	0.9920	0.6161	0.068*	
H6C	−0.5575	1.1256	0.5589	0.068*	

### Atomic displacement parameters (Å^2^)

**Table:**

	*U*^11^	*U*^22^	*U*^33^	*U*^12^	*U*^13^	*U*^23^
Ni1	0.02348 (10)	0.02426 (11)	0.03072 (11)	−0.00044 (11)	−0.00046 (11)	−0.00142 (9)
N1	0.0458 (11)	0.0336 (10)	0.0371 (9)	−0.0091 (8)	−0.0039 (10)	−0.0018 (9)
C1	0.0340 (9)	0.0322 (11)	0.0258 (9)	−0.0008 (9)	−0.0008 (7)	0.0003 (8)
S1	0.0523 (3)	0.0278 (3)	0.0333 (2)	−0.0104 (3)	−0.0001 (2)	0.00087 (19)
N2	0.0288 (7)	0.0434 (9)	0.0346 (9)	0.0041 (9)	0.0021 (10)	−0.0025 (7)
C2	0.0318 (9)	0.0280 (10)	0.0252 (8)	−0.0010 (8)	−0.0010 (7)	−0.0013 (7)
S2	0.0262 (2)	0.0527 (3)	0.0343 (2)	0.0089 (3)	0.0010 (2)	0.0033 (2)
N3	0.0339 (8)	0.0314 (10)	0.0369 (9)	−0.0033 (7)	0.0016 (9)	−0.0011 (9)
C3	0.0351 (9)	0.0332 (11)	0.0322 (9)	−0.0013 (8)	−0.0007 (9)	0.0025 (9)
C4	0.0585 (15)	0.0327 (12)	0.0492 (13)	−0.0105 (11)	−0.0028 (14)	−0.0001 (12)
N4	0.0334 (9)	0.0379 (10)	0.0382 (9)	0.0053 (8)	−0.0016 (9)	−0.0003 (9)
C5	0.0333 (10)	0.0368 (10)	0.0332 (9)	−0.0002 (9)	0.0007 (10)	−0.0034 (9)
C6	0.0326 (10)	0.0485 (13)	0.0547 (14)	0.0061 (10)	0.0018 (12)	−0.0008 (13)

### Geometric parameters (Å, °)

**Table:**

Ni1—N1	2.0210 (19)	S2—Ni1^iv^	2.5305 (6)
Ni1—N2	2.0231 (18)	N3—C3	1.131 (3)
Ni1—N4	2.0685 (19)	C3—C4	1.454 (3)
Ni1—N3	2.0782 (18)	C4—H4A	0.9600
Ni1—S2^i^	2.5305 (6)	C4—H4B	0.9600
Ni1—S1^ii^	2.5341 (6)	C4—H4C	0.9600
N1—C1	1.154 (3)	N4—C5	1.130 (3)
C1—S1	1.643 (2)	C5—C6	1.449 (3)
S1—Ni1^iii^	2.5341 (6)	C6—H6A	0.9600
N2—C2	1.149 (3)	C6—H6B	0.9600
C2—S2	1.641 (2)	C6—H6C	0.9600
			
N1—Ni1—N2	91.02 (8)	N2—C2—S2	178.0 (2)
N1—Ni1—N4	89.02 (8)	C2—S2—Ni1^iv^	100.02 (7)
N2—Ni1—N4	178.89 (9)	C3—N3—Ni1	173.7 (2)
N1—Ni1—N3	178.69 (9)	N3—C3—C4	178.7 (3)
N2—Ni1—N3	90.23 (8)	C3—C4—H4A	109.5
N4—Ni1—N3	89.73 (8)	C3—C4—H4B	109.5
N1—Ni1—S2^i^	90.09 (6)	H4A—C4—H4B	109.5
N2—Ni1—S2^i^	89.40 (5)	C3—C4—H4C	109.5
N4—Ni1—S2^i^	89.50 (6)	H4A—C4—H4C	109.5
N3—Ni1—S2^i^	90.29 (6)	H4B—C4—H4C	109.5
N1—Ni1—S1^ii^	90.35 (6)	C5—N4—Ni1	173.2 (2)
N2—Ni1—S1^ii^	93.23 (6)	N4—C5—C6	179.2 (3)
N4—Ni1—S1^ii^	87.88 (6)	C5—C6—H6A	109.5
N3—Ni1—S1^ii^	89.22 (6)	C5—C6—H6B	109.5
S2^i^—Ni1—S1^ii^	177.34 (2)	H6A—C6—H6B	109.5
C1—N1—Ni1	174.6 (2)	C5—C6—H6C	109.5
N1—C1—S1	178.77 (19)	H6A—C6—H6C	109.5
C1—S1—Ni1^iii^	98.29 (7)	H6B—C6—H6C	109.5
C2—N2—Ni1	170.90 (18)		

Symmetry codes: (i) *x*−1/2, −*y*+3/2, −*z*+1; (ii) −*x*, *y*−1/2, −*z*+3/2; (iii) −*x*, *y*+1/2, −*z*+3/2; (iv) *x*+1/2, −*y*+3/2, −*z*+1.
